# Melatonin and oxidative DNA damage in REM- and NREM-related obstructive sleep apnea: an exploratory analysis of sleep-stage phenotypes

**DOI:** 10.1007/s00405-026-10350-5

**Published:** 2026-06-01

**Authors:** Ercan Saruhan, Emre Ispir, Dilek Aslan Öztürk, Erdim Sertoglu, Yasemin Unal, Semai Bek, Gulnihal Kutlu

**Affiliations:** 1 Department of Biochemistry, Faculty of Medicine, Mugla Sitki Kocman University, Mugla, Turkey; 2 Department of Biochemistry, Izmir City Hospital, Izmir, Turkey; 3 Department of Neurology, Denizli State Hospital, Denizli, Turkey; 4 Department of Biochemistry, Gulhane Faculty of Medicine, University of Health Sciences, Ankara, Turkey; 5 Department of Neurology, Faculty of Medicine, Bandırma Onyedi Eylul University, Balikesir, Turkey; 6 Department of Neurology, Faculty of Medicine, Baskent University, Ankara, Turkey; 7 Department of Neurology, Faculty of Medicine, Mugla Sitki Kocman University, Mugla, Turkey

**Keywords:** Obstructive sleep apnea, Melatonin, 8-OHdG, Antioxidant

## Abstract

**Purpose:**

Obstructive sleep apnea is associated with intermittent hypoxia and oxidative stress. Whether oxidative and antioxidant biomarkers differ between rapid eye movement–related and non–rapid eye movement–related phenotypes remains unclear. We compared circulating melatonin and oxidative stress markers across sleep-stage phenotypes and disease severity categories.

**Methods:**

In this prospective case–control study, 165 participants (34 controls, 31 rapid eye movement–related obstructive sleep apnea, 100 non–rapid eye movement–related obstructive sleep apnea) underwent overnight polysomnography. Serum melatonin, 8-hydroxy-2′-deoxyguanosine, total oxidant status, and total antioxidant status were measured using enzyme-linked immunosorbent assay and colorimetric methods. The oxidative stress index was calculated as the ratio of total oxidant status to total antioxidant status multiplied by 100. Group comparisons were performed using nonparametric tests with appropriate post-hoc analyses. Additional analyses adjusted for age, body mass index, and sex using rank-transformed analysis of covariance. Correlations were evaluated using Spearman analysis with correction for multiple testing and formally compared between groups.

**Results:**

In unadjusted analyses, melatonin and 8-hydroxy-2′-deoxyguanosine levels were lower in obstructive sleep apnea, particularly in non–rapid eye movement–related and moderate disease. However, these differences were no longer statistically significant after adjustment for body mass index and sex. Total oxidant status, total antioxidant status, and oxidative stress index did not differ significantly between groups. Descriptive differences in correlation patterns were observed, but formal comparison showed no statistically significant between-phenotype differences.

**Conclusion:**

Circulating redox biomarkers in obstructive sleep apnea show modest differences that appear largely influenced by body mass index and sex. These findings should be considered exploratory and hypothesis-generating rather than evidence of phenotype-specific biological mechanisms.

## Introduction

Obstructive sleep apnea (OSA) is a prevalent disorder characterized by recurrent obstruction of the upper airway during sleep, leading to reduced blood oxygen saturation. These events cause hypoxemia and subsequently activate sympathetic nervous system [1, 2]. Rapid eye movement (REM)-related OSA is a phenotypic variant of OSA defined by the predominance of apneas and hypopneas during REM sleep [3, 4]. Although respiratory events can occur in both REM and non-REM (NREM) sleep, increased sympathetic activity during REM sleep further reduces the muscular tone of the pharyngeal muscles and substantially increases airway collapsibility [5, 6]. Consequently, patients with REM-related OSA are more vulnerable to respiratory distress during REM sleep. However, because REM sleep constitutes a smaller proportion of total sleep time, these patients often present with milder overall OSA severity based on the apnea–hypopnea index (AHI) [5, 6].

Differences in endocrine and sympathetic output between REM and NREM sleep may influence oxidative stress levels and the risk of related comorbidities, including cardiovascular disease and diabetes [7, 8]. Oxidative stress, defined as an imbalance between free radical production and antioxidant defense mechanisms, is a key pathophysiological mechanism in OSA [9]. Reactive oxygen species can modify the guanine base in DNA, forming 8-hydroxydeoxyguanosine (8-OHdG), a recognized marker of oxidative DNA damage [10]. Previous studies have demonstrated reduced antioxidant levels (e.g., vitamin A, vitamin E, and selenium) in OSA patients compared with healthy controls, suggesting that increased oxidative stress contributes to the development and progression of the disorder [9, 11–13].

Melatonin (MT) is a lipophilic neurohormone synthesized from tryptophan and secreted by the pineal gland in a circadian rhythm. Beyond its role in regulating the sleep–wake cycle, MT acts as a potent free radical scavenger and stimulates antioxidant enzymes such as glutathione peroxidase and superoxide dismutase [14, 15].

In this study, we aimed to investigate biomarkers of antioxidant capacity and oxidative stress in patients with REM-related OSA and compare them with those in patients with NREM-related OSA and healthy controls. This comparison was designed to elucidate the mechanisms of oxidative stress and antioxidant response across different OSA phenotypes. We aimed to explore whether oxidative and antioxidant biomarkers differ across REM-related and NREM-related OSA phenotypes.

## Materials and methods

### Study design

The Clinical Research Ethics Committee of Muğla Sıtkı Koçman University reviewed and approved this study (08/06/2017–11/II). The study was conducted in accordance with the ethical standards of the 1964 Declaration of Helsinki and its later amendments. Written informed consent was obtained from all participants.

We conducted a prospective case–control study including patients referred to the sleep laboratory of Muğla Sıtkı Koçman University Hospital between April 2019 and January 2020 for evaluation of OSA symptoms such as snoring, excessive daytime sleepiness, and witnessed apnea. Exclusion criteria were: age < 18 or > 70 years, body mass index (BMI) > 40 kg/m^2^, use of continuous positive airway pressure (CPAP), a history of other sleep disorders, and chronic systemic diseases such as diabetes mellitus, hypertension, and chronic obstructive pulmonary disease.

Demographic information such as sex, age, and BMI was obtained from medical records. Excessive daytime sleepiness was assessed using the ESS.

### Polysomnography

Overnight polysomnography (PSG) included continuous recordings of electroencephalogram, electrooculogram, electromyogram, nasal airflow, body position, and blood oxygen saturation. Data were scored manually by a certified neurologist according to the American Academy of Sleep Medicine (AASM) Manual [16]. PSG was performed during spontaneous sleep by a trained technician using an Embla N7000 system (Natus, Kanata, Canada). The AHI was defined as the number of apneas and hypopneas per hour of sleep. AHI_REM and AHI_NREM were calculated similarly for REM and NREM sleep periods. For severity-based analyses, OSA was further categorized according to AHI as mild (5–15 events/h), moderate (16–30 events/h), and severe (> 30 events/h), consistent with standard clinical thresholds.

Participants were divided into three groups based on their polysomnographic findings. The control group consisted of subjects with an AHI < 5, without complaints of excessive daytime sleepiness or snoring, and with an Epworth Sleepiness Scale (ESS) score < 10. REM-related OSA was defined as AHI ≥ 5, an AHI_REM/AHI_NREM ratio ≥ 2, and AHI_NREM < 15, according to the strict criteria of previous studies [3, 17]. Patients with AHI ≥ 5 who did not meet all criteria for REM-related OSA were classified as having NREM-related OSA.

### Biochemical analysis

After PSG, fasting venous blood samples were collected between 06:00 and 07:00 to minimize the influence of diurnal variations in melatonin levels. Samples were drawn into gel-separator tubes, allowed to clot at room temperature for 10–20 min, and centrifuged at 2000 × g for 10 min. Serum aliquots were stored at –80 °C until analysis.

Serum melatonin (Cat# E1013Hu) and 8-OHdG (Cat# E1436Hu) concentrations were measured using human-specific enzyme-linked immunosorbent assays (ELISA) (BT-Laboratory, Shanghai, China) according to the manufacturer’s instructions. The melatonin assay sensitivity was 2.51 ng/L, with a range of 5–1000 ng/L, and inter-assay CV < 10% and intra-assay CV < 8%. The 8-OHdG assay sensitivity was 0.25 ng/mL, with a range of 0.5–100 ng/mL, and inter-assay CV < 10% and intra-assay CV < 8%.

Serum total oxidant status (TOS) and total antioxidant status (TAS) were measured using commercial kits (Rel Assay Diagnostics, Ankara, Turkey). The oxidative stress index (OSI) was calculated as: OSI = [TOS (µmol/L)/TAS (µmol/L)] × 100.

### Statistical analysis

Statistical analyses were performed using IBM SPSS Statistics for Windows, Version 22.0 (IBM Corp., Armonk, NY, USA) and jamovi (version 2.6.26; The jamovi project, 2024; retrieved from https://www.jamovi.org). Descriptive statistics were expressed as minimum, maximum, median with interquartile range (IQR), or mean ± standard deviation, as appropriate. The Shapiro–Wilk test was used to assess normality of distributions. Between-group comparisons were conducted using the Kruskal–Wallis test, followed by the Dwass–Steel–Critchlow–Fligner post-hoc test when appropriate. Correlations between variables were evaluated using Spearman’s correlation coefficient. A two-sided p value < 0.05 was considered statistically significant.

Because age, BMI, and sex distribution differed across groups, additional analyses were performed using analysis of covariance (ANCOVA) in jamovi to adjust for these potential confounders. Given deviations from normality in several biomarkers (confirmed by Shapiro–Wilk test), rank-transformed ANCOVA models were applied as a sensitivity analysis. Adjusted marginal means were estimated for each group.

Selected analyses and data visualizations were independently reproduced using Python (version 3.x) to ensure analytical transparency and reproducibility. Python-based analyses were performed using the pandas, seaborn, matplotlib, scipy, and statsmodels libraries. Box-and-whisker plots were generated using median and interquartile ranges, with whiskers extending to 1.5 × IQR, and individual observations overlaid. Correlation heatmaps were constructed using Spearman correlation coefficients with Bonferroni-adjusted significance annotations. Python-derived outputs were cross-validated against SPSS results and yielded concordant findings.

## Results

A total of 165 participants were included in the study (54 female, 32.7%; 111 male, 67.3%), with a mean age of 44.1 ± 11.6 years (range, 18–67 years). Of these, 131 subjects were diagnosed with OSA (31 REM-related OSA, 23.7%; 100 NREM-related OSA, 76.3%), and 34 were healthy controls (AHI < 5). OSA patients were older and had higher BMI values compared with controls, and the proportion of males was higher in the NREM-related OSA group. Demographic and polysomnographic characteristics are summarized in Table 1.

**Table 1:** Demographic and polysomnographic data of patients and controls

	Control	REM-related OSA	NREM-related OSA
Subjects (n)	34	31	100
Gender Male, n (%) Female, n (%)	15 (44.1%) 19 (55.9%)	16 (51.6%) 15 (48.4%)	80 (80%) 20 (20%)
Age in years	34.9 ± 10.7	44.9 ± 11.1	46.9 ± 10.4
BMI in kg/m2	24.6 ± 5.1	29.5 ± 5.0	30.9 ± 5.2
ESS Score	4 (2- 9)	4 (2- 8)	7 (4- 10)
AHI	1.9 (0.8–3.5)	13.0 (9.3–15.9)	33.6 (20.4–54.5)
AHIREM	2.1 (0.7–7.7)	33.1 (22.2–40.0)	33.3 (15.2–57.2)
AHINREM	1.4 (0.6- 2.7)	8.8 (5.1–11.5)	33.3 (19.5–56.2)

Data are presented as mean ± SD for normally distributed variables and as median and quartiles (25th–75th percentiles) for non-normally distributed variables. BMI: body mass index, ESS: Epworth Sleepiness Scale, SD: standard deviation, AHI: apnea–hypopnea index

### Group comparisons of oxidative and antioxidant markers

**Fig. 1:**
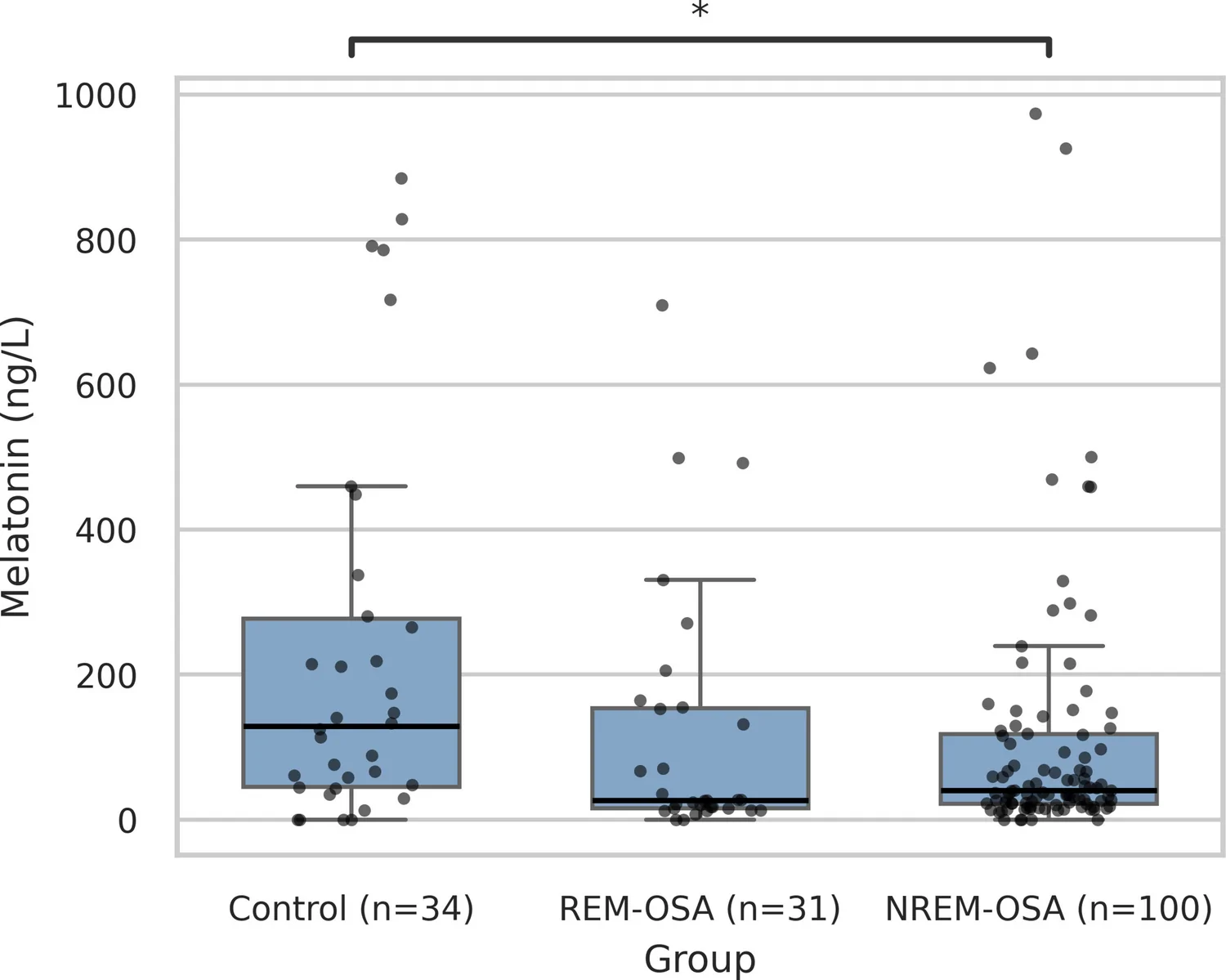
Serum Melatonin levels between REM‐phenotype groups Box-and-whisker plots (median, IQR; whiskers = 1.5 × IQR) with individual participants overlaid (grey dots). Groups: Control, REM-OSA, NREM-OSA. Asterisk bracket shows the significant pairwise comparison from Kruskal–Wallis with Dwass–Steel–Critchlow–Fligner (DSCF) post-hoc (Control vs NREM-OSA, p = 0.012). The number of participants per group is shown on the x-axis (n =). Abbreviations: MT, melatonin

Serum melatonin levels differed significantly among the three groups in unadjusted analyses (p = 0.009). Post-hoc analysis using the Dwass–Steel–Critchlow–Fligner test revealed significantly lower melatonin concentrations in the NREM-related OSA group compared with controls (p = 0.01). The difference between controls and REM-related OSA was borderline significant (p = 0.05), and no significant differences were observed between the two OSA subtypes (p = 0.652) (Fig. 1).

**Fig. 2:**
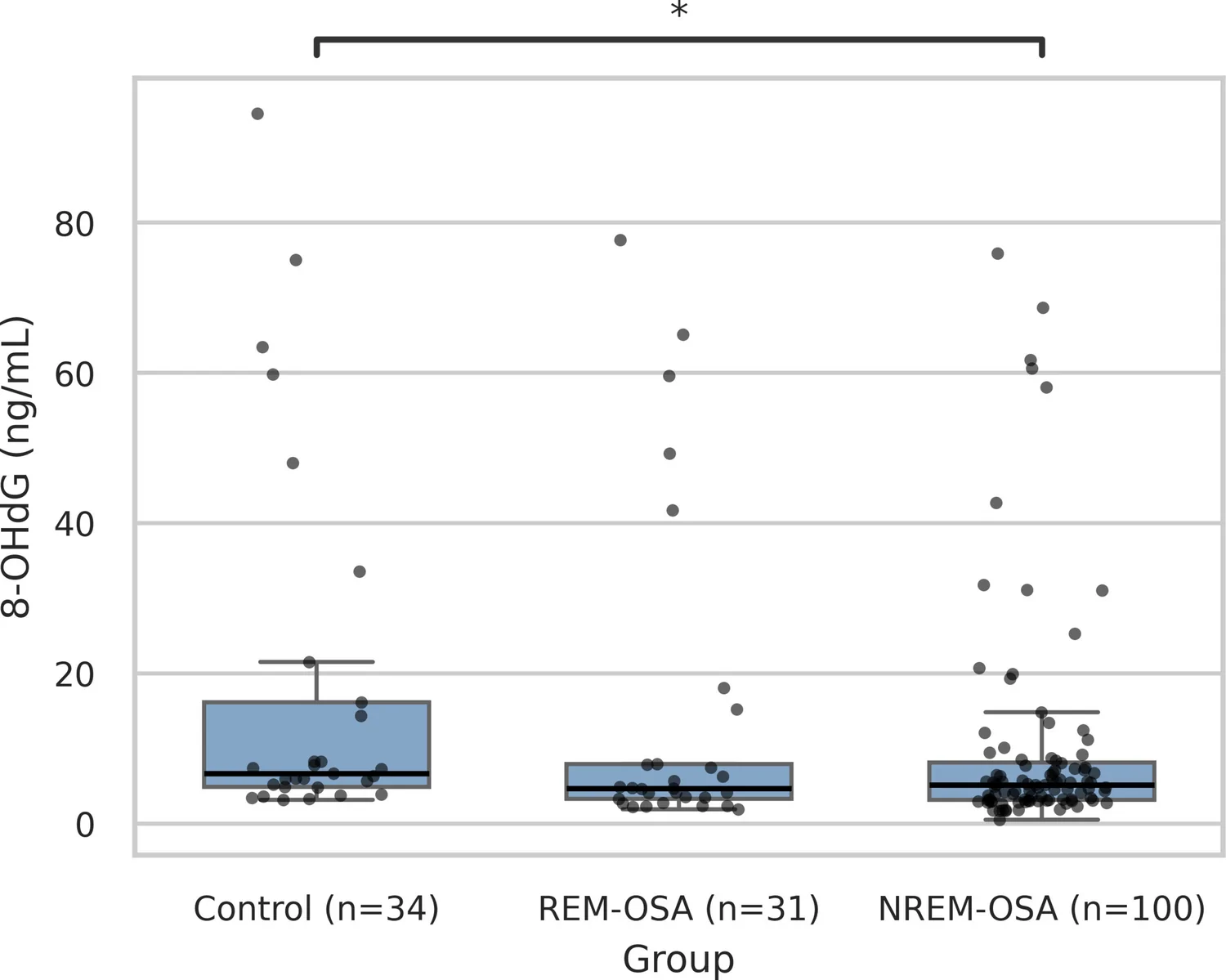
Serum 8-OHdG levels between REM‐phenotype groups. Box-and-whisker plots as in Fig. 1 with raw data points. Significant pairwise difference (Control vs NREM-OSA) is indicated (DSCF post-hoc, p = 0.020). Abbreviations: 8-OHdG, 8-hydroxy-2'-deoxyguanosine.

Similarly, serum 8-OHdG levels showed significant overall differences among groups (p = 0.03). Post-hoc testing indicated that NREM-related OSA patients had significantly lower 8-OHdG levels than controls (p = 0.02), while no significant differences were observed between controls and REM-related OSA or between the two OSA subgroups (Fig. 2).

In contrast, no statistically significant differences were detected among groups in TAS, TOS, or OSI values in unadjusted analyses (p = 0.240, p = 0.168, and p = 0.272, respectively) (Table 2).

**Table 2:** Comparison of antioxidant and oxidative stress biomarker levels between patients and controls

	Control	REM-related OSA	NREM-related OSA	p value *	p value (Adjusted)†
Subjects (n)	34	31	100		
Melatonin (ng/L)	143.8 (61.1, 337.3)	27.4 (17.5, 154.6)	43.6 (23.2, 122.9)	0.009	0.123
8-OHdG (ng/mL)	6.71 (4.88, 16.14)	4.68 (3.32, 7.97)	5.16 (3.15, 8.23)	0.027	0.640
TAS (mmol/L)	1.58 (1.42, 1.84)	1.44 (1.26, 1.66)	1.50 (1.29, 1.92)	0.240	0.069
TOS (µmol/L)	15.6 (13.39, 22.29)	14.59 (9.73, 23.94)	14.73 (10.51, 21.38)	0.168	0.693
OSI	10.21 (7.99, 13.48)	10.86 (7.07, 15.78)	9.52 (7.42, 12.55)	0.272	0.946

Data are presented as median (25th–75th percentile)

TAS: total antioxidant status; TOS: total oxidant status; OSI: oxidative stress index; 8-OHdG: 8-hydroxy-2′-deoxyguanosine

^*^ Kruskal Wallis test, Bold p values are indicating statistically differences between groups

^†^Adjusted p-values derived from rank-transformed ANCOVA models including age, body mass index (BMI), and sex as covariates

Bold values indicate statistical significance (p < 0.05)

After adjustment for age, BMI, and sex using rank-transformed ANCOVA models, the overall group effect for melatonin was no longer statistically significant (p = 0.123). BMI (p = 0.023) and sex (p = 0.001) remained independently associated with melatonin levels. Similarly, the adjusted group effect for 8-OHdG was not significant (p = 0.640), whereas BMI (p = 0.035) and sex (p = 0.007) were significant covariates. No significant adjusted group effects were observed for TOS or OSI. These findings indicate that the observed unadjusted differences in melatonin and 8-OHdG were largely attenuated after accounting for age, BMI, and sex.

### Subgroup analysis by OSA severity

**Fig. 3:**
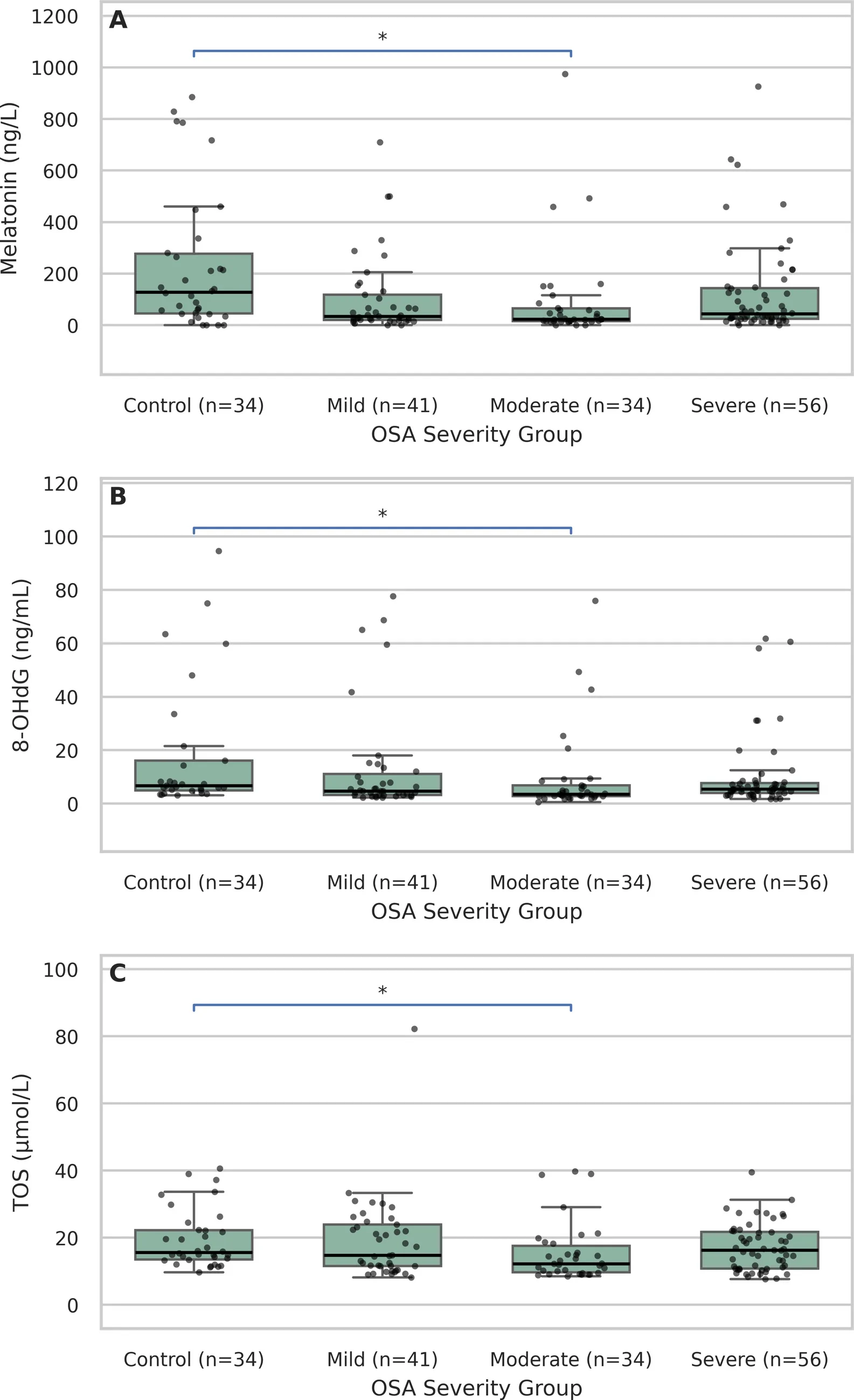
Serum oxidative/antioxidant markers in OSA severity. Stacked panels: A, Melatonin (MT); B, 8-OHdG; C, Total Oxidant Status (TOS). Box-and-whisker plots (median, IQR; whiskers = 1.5 × IQR) with individual points. OSA severity groups are ordered as Control, Mild, Moderate, Severe (based on AHI). Brackets show the significant/borderline post-hoc contrasts from Kruskal–Wallis with DSCF: Control vs Moderate OSA—MT (p = 0.014), 8-OHdG (p = 0.010), and TOS (p = 0.027). Group sizes (n =) are printed on the x-axis

When participants were stratified by AHI into mild (5–15 events/h), moderate (16–30 events/h), and severe (> 30 events/h) categories, unadjusted analyses demonstrated significant differences in serum melatonin (p = 0.007) and 8-OHdG levels (p = 0.010) (Table 3). Pairwise comparisons revealed that moderate OSA patients had significantly lower serum melatonin and 8-OHdG levels compared with healthy controls (p = 0.014 and p = 0.010, respectively) (Fig. 3A,B). TOS values also differed across severity groups (p = 0.027), although without a consistent monotonic trend. TAS values remained comparable among all severity categories in unadjusted analyses (p = 0.481) (Fig. 3C).

**Table 3:** Comparison of oxidative stress and antioxidant parameters across OSA severity groups

Parameter	Control (n = 34)	Mild (n = 41)	Moderate (n = 34)	Severe (n = 56)	p value*	p value (Adjusted)†
Melatonin (ng/L)	129 (54–295)	35.1 (0–188)	23.7 (0–216)	45.0 (0–205)	0.007	0.072
8-OHdG (ng/mL)	6.71 (3.6–13.2)	4.76 (2.7–9.4)	3.58 (1.2–8.9)	5.51 (2.1–10.8)	0.010	0.117
TAS (µmol/L)	1.58 (1.43–1.77)	1.55 (1.37–1.74)	1.44 (1.30–1.66)	1.54 (1.38–1.70)	0.481	0.018
TOS (µmol/L)	15.6 (11.9–19.8)	14.8 (10.2–19.1)	12.2 (9.4–17.8)	16.3 (11.7–20.5)	0.027	0.206

Data are presented as median and quartiles (25th–75th percentiles). 8-OHdG: 8-Hydroxydeoxyguanosine, TAS: total antioxidant status, TOS: total oxidant status

^*^Kruskal Wallis test, Bold values indicate statistical significance (p < 0.05)

^†^Adjusted p-values derived from rank-transformed ANCOVA models including age, BMI, and sex as covariates

In ANCOVA models adjusting for age, BMI, and sex, the overall severity-group effect for melatonin was attenuated (p = 0.072), and the effect for 8-OHdG was no longer statistically significant (p = 0.117). In contrast, TAS levels were significantly lower in moderate (p = 0.018) and severe OSA (p = 0.034) compared with controls after adjustment. No significant adjusted group effects were observed for TOS or OSI.

### Correlation analysis

**Fig. 4:**
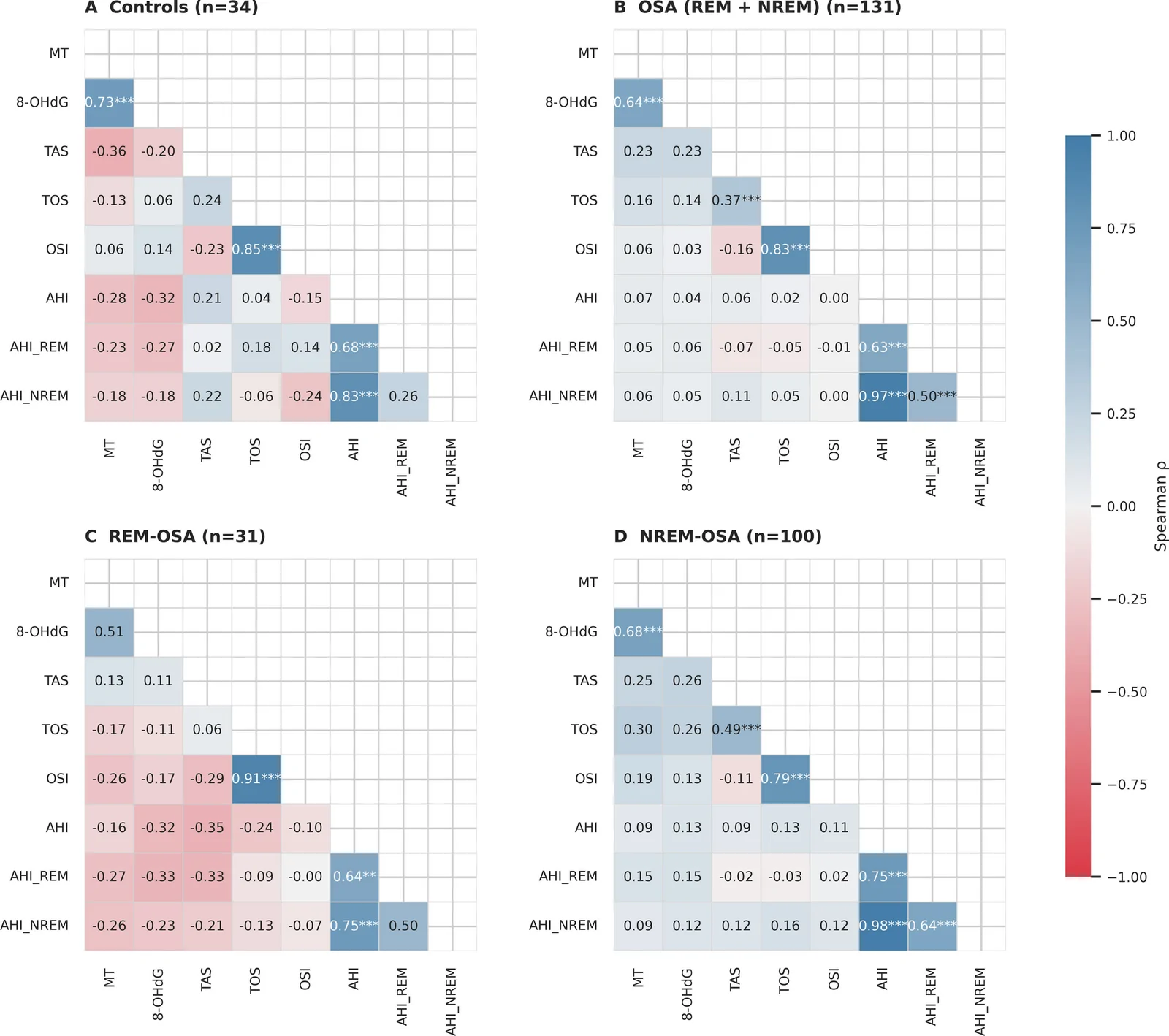
Correlation structure of oxidative, antioxidant, and apnea indices. Heatmaps show pairwise Spearman correlation coefficients (ρ) among MT, 8-OHdG, TAS, TOS, OSI, AHI, AHI_REM, and AHI_NREM. Panels: A, Controls; B, OSA pooled (REM + NREM); C, REM-OSA; D, NREM-OSA. Sample sizes (n) are indicated in each panel title. Cells are annotated with ρ and significance stars after Bonferroni correction for multiple testing: *** p < 0.001, ** p < 0.01, * p < 0.05; blanks indicate non-significant correlations. A single shared color scale (ρ range − 1 to + 1; blue = positive, red = negative) is used across panels to allow direct visual comparison

Correlation heatmaps are presented in Fig. 4. In controls, a strong positive correlation was observed between melatonin and 8-OHdG (ρ = 0.73, p < 0.001), which remained significant after Bonferroni correction. In NREM-related OSA, significant correlations were noted between melatonin and 8-OHdG (ρ = 0.68, p < 0.001) and between TAS and TOS (ρ = 0.486, p < 0.001). In the REM-related OSA group, no correlations remained significant after Bonferroni correction, although a moderate melatonin–8-OHdG association was observed before correction (ρ = 0.51, unadjusted p = 0.016).

To formally assess whether correlation patterns differed between REM-related and NREM-related OSA groups, Fisher’s r-to-z transformation was applied to compare correlation coefficients, with false discovery rate correction for multiple testing. After correction, no statistically significant differences were observed between REM and NREM groups for oxidative or antioxidant biomarker correlations. The only significant difference was detected for the structural association between AHI and AHI_NREM, reflecting their mathematical interdependence rather than a biological distinction.

Accordingly, while visual inspection of correlation heatmaps suggests potential differences in redox coupling patterns, these findings should be interpreted as exploratory and hypothesis-generating rather than definitive evidence of distinct biological phenotypes.

## Discussion

This study compared oxidative and antioxidant biomarkers among patients with REM-related and NREM-related OSA and healthy controls. In unadjusted analyses, melatonin and 8-OHdG levels were lower in OSA—particularly in the NREM subgroup—while TAS, TOS, and OSI showed limited group differences. However, after adjustment for age, BMI, and sex using rank-transformed ANCOVA models, group differences in melatonin and 8-OHdG were no longer statistically significant. Instead, BMI and sex emerged as significant independent predictors of these biomarkers. These findings suggest that demographic and anthropometric factors substantially contribute to the observed unadjusted differences and that sleep-stage phenotype alone does not independently explain variations in circulating melatonin or oxidative DNA damage markers.

The reduction of melatonin observed in unadjusted analyses is consistent with its established role in sleep regulation and antioxidant defense, and prior studies have reported altered nocturnal melatonin secretion and circadian disruption in OSA. Barnas et al. demonstrated significantly lower nocturnal serum melatonin concentrations in patients with OSA compared with controls (2 AM: 68 vs 109 pg/mL; 6 AM: 41 vs 68 pg/mL) and reported altered circadian secretion patterns in approximately one-quarter of patients [18]. Importantly, in that cohort, patients with OSA were significantly older and had higher BMI values than controls. Given that melatonin secretion declines with age and has been shown to correlate inversely with body mass, part of the observed difference in melatonin levels may have been influenced by demographic and anthropometric disparities between groups. This consideration parallels our own findings, in which adjustment for age, BMI, and sex attenuated the between-group differences in melatonin levels.

Consistent with the broader literature, Karel et al. reported a disease-severity–dependent reduction in 24-h salivary melatonin secretion among 169 OSA patients, including lower peak concentrations and reduced total melatonin exposure compared with reference individuals [19]. Notably, these alterations persisted after adjustment for age and sex, suggesting that OSA itself may contribute to circadian dysregulation in certain populations. Together, these findings indicate that melatonin suppression may occur in OSA, but its magnitude and independence from anthropometric factors likely vary across cohorts. Our results suggest that, in stage-specific comparisons, demographic factors may partly account for observed differences in circulating melatonin concentrations.

Notably, 8-OHdG levels were lower in NREM-related OSA than in controls in unadjusted comparisons. This finding appears counterintuitive, as intermittent hypoxia would be expected to increase oxidative DNA damage. However, 8-OHdG reflects not only the rate of DNA oxidation but also the efficiency of repair and clearance mechanisms. Thus, circulating concentrations may represent the net balance between oxidative injury and DNA repair activity rather than the absolute burden of oxidative damage. Previous studies provide support for this interpretation. Bhatti et al. demonstrated that melatonin suppression during daytime sleep was associated with reduced urinary 8-OHdG excretion, suggesting impaired DNA repair, whereas Zanif et al. reported increased 8-OHdG excretion following melatonin supplementation, consistent with enhanced oxidative lesion repair [20, 21]. Taken together, these observations raise the possibility that alterations in melatonin physiology may influence oxidative DNA repair dynamics in OSA. Alternative explanations should also be considered. Circulating 8-OHdG levels measured by ELISA may be influenced by assay variability, renal clearance, and systemic metabolism. In addition, early morning sampling and circadian fluctuations may affect measured concentrations, and serum levels may not directly reflect tissue-level oxidative stress. Therefore, the lower 8-OHdG levels observed in our cohort should be interpreted cautiously, and causal mechanisms cannot be confirmed from this cross-sectional dataset.

However, because the group differences in both melatonin and 8-OHdG were attenuated after adjustment for BMI and sex, this interpretation should be considered hypothesis-generating rather than mechanistically confirmed. This interpretation is further supported by a large cross-sectional study by Peres et al., who evaluated circulating oxidative stress biomarkers in 402 patients undergoing polysomnography [22]. Although OSA severity was independently associated with 8-isoprostane, AHI was not an independent predictor of 8-OHdG after adjustment for age, sex, BMI, smoking status, cardiovascular disease, and statin use [22]. These findings suggest that DNA oxidation markers such as 8-OHdG may be more strongly influenced by demographic and metabolic factors than by OSA severity per se, consistent with our adjusted results.

In severity-based analyses, TAS levels were significantly lower in moderate and severe OSA compared with controls after adjustment for age, BMI, and sex, representing the only biomarker that remained significant. This finding suggests that global antioxidant capacity may be modestly reduced with increasing OSA burden, independent of demographic confounding. Supporting this interpretation, Christou et al. reported no overall difference in antioxidant capacity between OSA patients and controls, but demonstrated a significant negative correlation between TAS and AHI in severe OSA [23]. Similarly, other studies have shown reductions in antioxidant capacity following nocturnal hypoxic exposure, although the magnitude and severity dependence of this effect appear variable across cohorts [24]. Together, these findings indicate that antioxidant depletion in OSA may become evident particularly at higher disease burden, but remains heterogeneous and context-dependent.

Previous studies evaluating other oxidative markers have similarly demonstrated heterogeneity in oxidative stress responses across disease severity. For example, elevated advanced oxidation protein products (AOPP) have been reported in moderate and severe OSA compared with controls, supporting a link between disease burden and oxidative imbalance, although AOPP was not measured in the present study [25]. Nevertheless, the degree to which such alterations are independent of obesity and other cardiometabolic confounders remains variable across studies.

Correlation analyses showed a positive association between melatonin and 8-OHdG in controls. In NREM-related OSA, melatonin–8-OHdG and TAS–TOS correlations remained significant after multiple-testing correction, whereas no significant correlations were observed in REM-related OSA. However, when the strength of these correlations was directly compared between REM- and NREM-related OSA using Fisher’s r-to-z transformation, no statistically significant differences were found. These findings suggest that although correlation patterns appeared different descriptively, we did not demonstrate a statistically confirmed difference between sleep-stage phenotypes. Therefore, these findings should be interpreted cautiously and considered exploratory rather than evidence of distinct biological phenotypes.

Several limitations should be acknowledged. First, the control and OSA groups were not fully matched for age, BMI, and sex and, the REM-related OSA subgroup was relatively small (n = 31), which may have limited statistical power, particularly for correlation analyses requiring correction for multiple comparisons. No formal a priori sample size calculation was performed because the study was exploratory and based on eligible participants recruited during the predefined study period. Therefore, the study may have been underpowered to detect small between-group differences, especially in subgroup and correlation analyses. The absence of significant associations after Bonferroni adjustment in this subgroup may therefore reflect limited power rather than true absence of biological coupling. No formal a priori power calculation was performed, and the multiple subgroup and correlation analyses increase the possibility of type I error despite correction procedures. Additionally, relatively small subgroup sizes, particularly in the REM-related OSA cohort, may increase the risk of type II error and limit the robustness of subgroup-specific findings.

Second, we employed a strict definition of REM-related OSA to ensure a clearly delineated REM-dominant phenotype. Although this approach improves internal validity and reduces misclassification, it may limit generalizability to studies using broader REM-OSA criteria.

Third, blood samples were collected between 06:00 and 07:00, a time when endogenous melatonin levels physiologically decline after their nocturnal peak. Although sampling time was standardized across all groups to ensure internal consistency, early morning measurements may underestimate peak nocturnal melatonin secretion. This single time-point measurement does not capture circadian dynamics and limits the ability to draw conclusions about phenotype-specific melatonin regulation. In addition, minor differences in wake time, sleep fragmentation, or early light exposure following polysomnography could influence circulating melatonin concentrations [23]. Therefore, while between-group comparisons remain valid due to uniform sampling conditions, absolute melatonin values should be interpreted in the context of circadian timing. Future studies incorporating serial nocturnal measurements or dim-light melatonin onset assessment would further clarify stage-specific circadian alterations in OSA.

## Conclusions

Overall, our findings suggest that oxidative and antioxidant alterations in OSA are modest and appear to be largely influenced by BMI and sex, with potential severity-dependent effects on global antioxidant capacity. Larger, longitudinal studies are needed to clarify the independent contribution of sleep-stage phenotype and to determine the clinical utility of these biomarkers in OSA.

## Data Availability

The datasets used and/or analyzed during the current study are available from the corresponding author on reasonable request.
